# Carbon Fixation from Industrial Flue Gas via CO_2_ Mineral Carbonation: Principles, Technical Advances, and Future Directions

**DOI:** 10.3390/ma18214935

**Published:** 2025-10-28

**Authors:** Yanli Sun, Xujiang Wang, Zhipeng Ma, Yanmei Cheng, Bingbing Xie, Mengning Liu, Jingwei Li, Chenggong Sun, Wenlong Wang

**Affiliations:** 1 National Engineering Laboratory for Reducing Emissions from Coal Combustion, Shandong University, Jinan 250061, China; 2 Shenzhen Research Institute of Shandong University, Shenzhen 518000, China; 3 School of Chemical Engineering, University of Birmingham, Birmingham B15 2TT, UK

**Keywords:** CO_2_ mineral carbonation, industrial flue gas, geological minerals, cement and concrete, solid waste

## Abstract

The predominant reliance on fossil fuel combustion for energy production continues to release significant amounts of CO_2_, causing global temperatures to approach the critical threshold of 1.5 °C. This has led to an increasing frequency of extreme weather events, which pose severe threats to ecosystems, economic development, and human safety. CO_2_ mineral carbonation, by mimicking the natural weathering process, facilitates the reaction between minerals and CO_2_, resulting in long-term and stable sequestration. Over several decades of development, this technology has evolved from its initial application in natural mineral sequestration to broader use in sectors such as cement concrete, industrial solid waste recycling, and chemical production. It offers an innovative solution for emission reduction and resource utilization in high-emission industries, particularly those characterized by difficult-to-decarbonize point sources. This technology holds promise for the on-site treatment and value-added utilization of industrial flue gas and solid waste at the source. This review systematically presents the research advancements and emerging trends in CO_2_ mineral carbonation, covering key aspects such as reaction mechanisms, research progress, engineering demonstrations, and prospects. A particular focus is placed on analyzing the principles of direct and indirect carbonation. The review synthesizes developments in three research domains—geological minerals, cement concrete, and industrial solid waste—and introduces several representative engineering demonstration projects. Furthermore, it discusses the challenges faced at the scientific, technological, and application levels and proposes future directions for the development of CO_2_ mineral carbonation technology. This review aims to provide a comprehensive reference for future research, fostering the continued innovation and commercialization of CO_2_ mineral carbonation.

## 1. Introduction

The continuous rise in atmospheric CO_2_ concentrations has become a key driver of global climate change and the increasing frequency of extreme weather events. In 2024, global energy-related carbon emissions reached a record high of 37.8 Gt, resulting in an increase in atmospheric CO_2_ concentrations to 422.5 ppm, the highest level ever recorded [1]. That year was confirmed as the hottest on record, with the global mean surface temperature approaching or potentially surpassing the 1.5 °C warming threshold set by the Paris Agreement. This warming has directly intensified the frequency and severity of extreme climate events, including droughts, heavy precipitation, and storms [2,3]. Recent studies indicate that climate change-induced extreme weather has caused an average annual economic loss of approximately USD 143 billion worldwide [4]. The relentless accumulation of CO_2_ emissions poses a profound threat to global ecosystems, economic development, and human security.

Although large-scale application of renewable energy and enhancement in energy efficiency are widely recognized as fundamental pathways to carbon mitigation, industrial processes (e.g., cement, steel, and chemical production) and fossil fuel utilization (e.g., coal-fired power generation) remain the dominant sources of CO_2_ emissions at the present stage, and their complete elimination in the short term is challenging. In 2024, the power sector accounted for 38.7% of global carbon emissions, while the industrial sector contributed 28.5%. The CO_2_ concentration in industrial flue gases varies considerably, typically ranging from 5% to 25%, and the gas composition is complex, often containing pollutants (for example, SO_x_ and NO_x_), which complicates mitigation efforts. Against this backdrop, carbon capture, utilization, and storage (CCUS) has emerged as a key technology for addressing the challenges mentioned above. By separating and capturing CO_2_ from industrial sources or directly from the air, succeeded by compression and transport, CCUS enables its permanent storage in geological formations or conversion into value-added products. This technology is widely regarded as indispensable for achieving deep decarbonization of energy systems. However, despite its considerable potential, large-scale deployment of CCUS still faces multiple challenges, including the high energy and cost intensity of capture processes, insufficient economic viability of large-scale transport and storage, limited efficiency in resource utilization, and underdeveloped business models and policy support frameworks.

CO_2_ mineral carbonation (CMC), as an important carbon capture and utilization (CCU) technology, is based on simulating the natural weathering of rocks. In this process, CO_2_ reacts with Ca- and Mg-containing alkaline oxides present in natural minerals or industrial solid waste to form stable and non-decomposable carbonates, thereby enabling long-term carbon fixation. Both industrial flue gases and alkaline solid waste hold significant potential for application in this context. Studies indicate that CO_2_ mineralization using alkaline solid waste could fix approximately 4.02 Gt of CO_2_ annually worldwide, corresponding to about 12.5% of global anthropogenic emissions. Among all countries, China exhibits the greatest potential, with the capacity to reduce its total annual carbon emissions by an estimated 19.2% [5]. Moreover, mineralization products can partially substitute for cement clinker or serve as aggregates in concrete, thereby indirectly reducing Portland cement consumption and further lowering CO_2_ emissions from the cement industry. Consequently, implementing CO_2_ mineralization with industrial flue gases and alkaline solid waste not only mitigates greenhouse gas emissions and alleviates the burden of solid waste accumulation but also contributes to achieving global carbon neutrality and advancing resource circularity [6].

Since Seifritz first proposed CMC sequestration in 1990, the technology has been regarded as a promising pathway for large-scale carbon storage with broad application potential [7]. Early studies primarily focused on naturally abundant Ca- and Mg-rich minerals like wollastonite, olivine, and serpentine, which can react with CO_2_ through mineralization to achieve carbon fixation. Theoretically, the use of natural minerals for CO_2_ storage offers enormous potential, with a maximum sequestration capacity exceeding 10^5^ Gt CO_2_ [8]. Although the CO_2_ mineralization process is thermodynamically favorable (negative Gibbs free energy), the reaction proceeds extremely slowly under natural conditions, often requiring hundreds to thousands of years. To accelerate the process at an engineering scale, natural minerals typically need to be ground and activated, followed by reactions conducted under elevated temperature and pressure, inevitably leading to high energy consumption and costs [9,10]. In contrast, alkaline solid waste, owing to their inherent alkalinity and relatively high reactivity, is considered more suitable for CO_2_ mineralization [11]. Such waste includes steelmaking slags, coal combustion by-products, fuel combustion residues, mining and mineral processing waste, incineration fly ash, cement and concrete waste, and pulp and paper residues [5]. These industries not only emit substantial amounts of alkaline solid waste during production but also release large volumes of industrial flue gases. Consequently, applying CO_2_ mineralization to alkaline solid waste offers the dual benefit of capturing CO_2_ from industrial emissions while simultaneously valorizing solid waste resources [12]. Over the past decade, CO_2_ mineralization technologies have advanced rapidly, with multiple technological pathways progressing to engineering-scale demonstrations and even commercial deployment. Current application directions include geological mineral sequestration, carbonation curing of cement-based materials, mineralization of recycled concrete, and CO_2_ utilization through industrial solid waste carbonation.

Notably, numerous studies have demonstrated that the carbonate products produced during the carbonation reaction may clog mineral pores or cover their surfaces, thereby hindering CO_2_ mass transfer and the leaching of Ca^2+^ and Mg^2+^ ions, ultimately restricting the continuation of the reaction. Consequently, simply accelerating the process by increasing CO_2_ concentration, pressure, or other reaction parameters may prove counterproductive. To ensure the sustainability of mineral carbonation, multiple parameters—including temperature, pressure, particle size, and CO_2_ concentration—should be considered in an integrated manner to identify optimal conditions that support a stable and continuous reaction process [9,13]. Furthermore, approaches such as mechanical ball milling, additive-assisted activation, and ultrasonic enhancement have been shown to effectively increase the specific surface area of reactants and improve interfacial mass transfer, thereby enhancing the efficiency of CO_2_ mineral sequestration.

To advance CO_2_ mineralization as a viable solution to climate change, a systematic synthesis and forward-looking perspective are required. This study provides a detailed analysis and comparison of the different reaction pathways and mechanisms of CO_2_ mineralization, offering an in-depth summary of its innovative progress across diverse application scenarios. Several representative industrial applications are also examined. Finally, the challenges and bottlenecks currently impeding the industrial deployment of CO_2_ mineralization technologies are discussed, along with proposed directions for future research and policy development. Through this work, we aim to provide theoretical insights and technical guidance for the large-scale application of CO_2_ mineralization, thereby supporting industrial carbon reduction and the advancement of negative-emission technologies.

## 2. Principles for CO_2_ Mineral Carbonation

The CMC reaction essentially involves the transformation of the acidic gas CO_2_ into stable carbonates through reactions with alkaline minerals, primarily Ca- and Mg-based phases. Its principal advantage lies in the permanence and security of carbon sequestration. However, this reaction generally requires overcoming substantial activation energy barriers, which results in relatively slow kinetics. Depending on the reaction pathway, conventional CMC can be categorized into two types: direct carbonation and indirect carbonation. To further enhance the reaction kinetics, emerging approaches such as physically assisted mineralization and enzyme-mediated biocatalysis are being actively developed, aiming to improve the carbonation rate and carbon fixation efficiency.

### 2.1. Direct Carbonation

**Figure 1:**
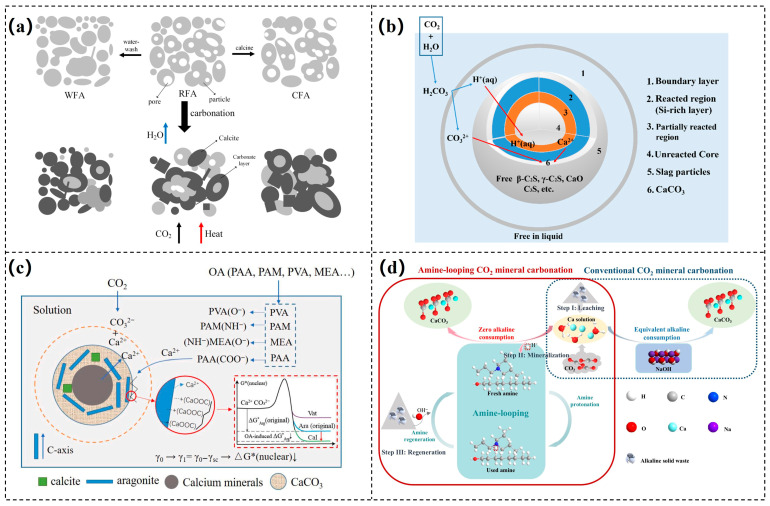
Different principles for CO_2_ mineral carbonation: (**a**) direct dry carbonation [16]; (**b**) direct wet carbonation [20]; (**c**) direct aqueous carbonation [21]; (**d**) indirect carbonation [22].

Direct carbonation refers to the direct reaction of alkaline minerals or their slurries with CO_2_, characterized by a relatively simple process and strong operational feasibility. In accordance with the liquid-to-solid (L/S) ratio, direct carbonation can be classified into dry carbonation, wet carbonation, and aqueous carbonation. Specifically, when it is lower than 0.2, the process is referred to as dry carbonation; values between 0.2 and 5 correspond to wet carbonation; and those exceeding 5 are defined as aqueous carbonation [14,15]. Direct dry carbonation, also known as gas-solid carbonation, involves the direct interaction and reaction of CO_2_ with Ca- and Mg-bearing minerals under low L/S ratios, as shown in Equation (1) and Figure 1a [16]. Due to limitations in diffusion efficiency, direct dry carbonation typically exhibits low conversion rates and sluggish reaction kinetics, often necessitating elevated temperature and pressure to accelerate the process [17,18]. However, as carbonation is an exothermic reaction, higher temperatures suppress the forward reaction thermodynamically [19]. Moreover, achieving suitable temperature, pressure, and particle size conditions requires substantial energy input, together with significant capital investment in equipment and high operational and maintenance costs, which collectively constrain the broader application and large-scale deployment of this technology.

(1) $$(\mathrm{Ca},\mathrm{Mg}{)}_{x}Si_{y}O_{x+2y+z}H_{2z}\left(s\right)+\mathrm{xC}O_{2}(g)\to x\left(\mathrm{Ca}/\mathrm{Mg}\right)CO_{3}\left(s\right)+\mathrm{ySi}O_{2}\left(s\right)+zH_{2}O\left(g/L\right)$$

When the L/S ratio exceeds 0.2, the carbonation process transitions from a simple gas–solid reaction to a gas–liquid–solid multiphase reaction. The involvement of water significantly enhances mass transfer efficiency, as some CO_2_, Ca^2+^ and Mg^2+^ can dissolve in water, facilitating the carbonation reaction. This process is often described using surface coverage models and shrinking core models, with Figure 1b illustrating the microscopic reaction mechanism based on the shrinking core model [20]. According to the shrinking core model, the reaction progresses from the particle surface inward, resulting in the gradual shrinking of the unreacted core. Simultaneously, the resulting carbonate products precipitate onto the particle surfaces, forming a product layer (also known as the passivation layer) [23,24]. This product layer impedes the diffusion of CO_2_, Ca^2+^, and Mg^2+^, leading to a slowdown in the carbonation reaction. In practical applications, this method is commonly used for the resource utilization of industrial solid waste and the preparation of building materials. Water exerts a critical effect in this process: within a certain range, increasing water content helps accelerate mineral dissolution and CO_2_ absorption, thereby promoting the carbonation reaction. However, when the water content exceeds a critical threshold, excess water can clog the material’s pores, limiting CO_2_ diffusion and thus decreasing the efficiency of the carbonation reaction [25].

The basic mechanism of direct solution carbonation is similar to that of wet carbonation, but with a higher L/S ratio. Mineral powders are dispersed in an aqueous solution to form a slurry. After CO_2_ dissolves, carbonic acid is formed, which further dissociates to produce H^+^ ions, leading to a decrease in pH and promoting the continuous leaching of Ca^2+^ and Mg^2+^ from the reaction materials (as shown in Equations (2) and (3)). These Ca^2+^ and Mg^2+^ then react with carbonate ions on the mineral surface to form carbonate precipitates [26,27]. Figure 1c illustrates the microscopic reaction mechanism of direct solution carbonation under the influence of different additives [21]. However, due to the inherent stability of Ca- and Mg-containing minerals in mildly acidic environments, the efficiency of ion leaching is limited, resulting in a relatively low overall mineralization conversion rate. To enhance the reaction efficiency, methods such as mechanical ball milling, ultrasonic activation, or the addition of specific activators are often employed to improve mass transfer. However, these measures are typically accompanied by increased energy consumption and higher costs [28].

(2) $$CO_{2}\left(g\right)+H_{2}O(L)\to H_{2}CO_{3}(\mathrm{aq})\to H^{+}+HCO_{3}^{-}\to H^{+}+CO_{3}^{2-}$$

(3) $$\left(Ca,Mg{)}_{x}Si_{y}O_{x+2y+z}H_{2z}(s)+2xH^{+}(aq)\to xCa^{2+}/Mg^{2+}(aq)+ySiO_{2}(s)+(x+z)H_{2}O(L\right)$$

### 2.2. Indirect Carbonation

Indirect carbonation typically divides the entire mineralization process into two stages: first, the extraction of Ca^2+^ and Mg^2+^ from alkaline minerals into the solution, as expressed in Equation (3); second, the reaction of Ca^2+^ and Mg^2+^ in the solution with CO_2_ under alkaline conditions to form carbonate precipitates (as shown in Equations (4) and (5)). This method is also referred to as the pH-swing method [29]. In the first stage, acid, base, or salt additives (usually under low pH conditions) are employed to facilitate the dissolution of Ca^2+^ and Mg^2+^ from the minerals. Subsequently, by adjusting the system to an alkaline environment, CO_2_ can react with the Ca^2+^ and Mg^2+^ in the solution, thus achieving carbonation fixation. The use of acidic reagents efficiently leaches Ca^2+^ and Mg^2+^, but the carbonation reaction stage requires the additional introduction of alkaline reagents to adjust the pH, which significantly increases the process cost [30]. In contrast, certain organic solvents, such as ammonium salts, exhibit strong chelating properties, achieving leaching efficiencies comparable to acidic reagents while offering better economic advantages. Figure 1d presents an indirect mineralization process mediated by an amine cycle, which demonstrates higher reaction rates and conversion degrees [22]. Although indirect carbonation can produce high-purity carbonate products, it still faces significant challenges, including difficulties in recovering additives, complex process flows, and cost control. Therefore, future research needs to continue exploring process optimization design and the development of low-cost, recyclable additives [31].

(4) $$CO_{2}(g)+2OH^{-}(\mathrm{aq})\to CO_{3}^{2-}(\mathrm{aq})+H_{2}O(L)$$

(5) $$Ca^{2+}/Mg^{2+}(\mathrm{aq})+CO_{3}^{2-}(\mathrm{aq})\to (\mathrm{Ca}/\mathrm{Mg})CO_{3}(s)$$

### 2.3. Emerging Technologies and Principles

The primary kinetic bottleneck of CMC arises from the high energy barrier for the leaching of Ca^2+^ and Mg^2+^, as well as the limited solubility of CO_2_ in the reaction system. To overcome these limitations, researchers have developed various enhancement techniques. Among these, physically assisted mineralization and biologically mediated mineralization are two promising technologies with significant application potential.

Physically assisted mineralization primarily relies on the introduction of external energy fields, such as ultrasound, microwaves, and mechanical forces, to improve the surface structure of minerals and mass transfer conditions, thereby significantly accelerating the carbonation reaction rate at the microscopic level. Notably, cavitation-enhanced ultrasound has attracted considerable attention due to its unique mechanism. The cavitation bubbles generated under ultrasound collapse, creating instantaneous high temperatures, high pressures, and intense micro-jets, which disrupt the mineral surface structure and increase the specific surface area to accelerate the leaching of Ca^2+^ and Mg^2+^, also significantly promote the dissolution and diffusion of CO_2_, thereby greatly improving the mineralization reaction efficiency.

Biologically mediated mineralization, on the other hand, relies on the effect of biological enzymes (such as carbonic anhydrase) to catalyze the hydration of CO_2_, facilitating the formation of HCO_3_^−^ and subsequently driving the reaction between Ca^2+^ and Mg^2+^ with carbonate ions to form stable carbonate precipitates. Compared to physical enhancement methods, biologically mediated processes can achieve efficient conversion under mild temperature, pressure, and pH conditions, providing benefits like low energy consumption and environmental friendliness. However, enzymes are highly sensitive to environmental factors such as temperature, pH, and ionic strength, leading to significant challenges regarding their stability and sustainability in complex systems and large-scale industrial applications.

Therefore, despite the significant reaction enhancement potential of both physically assisted and biologically mediated mineralization, key issues such as energy consumption, cost, and system stability must still be addressed for their industrial deployment.

### 2.4. Physical–Chemical Characterization of CO_2_ Mineral Carbonation

Characterization techniques provide essential support for understanding and optimizing the reaction mechanisms of CMC technology. SEM-EDX enables detailed examination of the morphology, microstructure, and elemental distribution of carbonation products. SEM offers high-resolution images that reveal surface topography, particle aggregation, and textural features, while EDX provides spatially resolved elemental analysis, facilitating the identification of carbonation phases and their distribution within the matrix [32]. XRD plays a crucial role in determining and elucidating the crystalline phase transformations involved in the mineral carbonation process. By confirming the formation of carbonate phases such as calcite and aragonite, XRD allows for the evaluation of product crystallinity and stability under various reaction conditions [32,33]. FTIR provides additional insights into chemical bonding within the carbonation products. FTIR can clearly detect the formation of carbonate functional groups and monitor the evolution of chemical bonds during carbonation [32]. Understanding these reaction kinetics and the influence of different reaction parameters on product formation is essential for elucidating the carbonation mechanism. Furthermore, TGA serves as a key tool for quantitatively assessing the extent and rate of carbonation by measuring mass changes during controlled heating [34]. ICP-MS enables trace element analysis, offering valuable information on the migration and retention behaviors of metals within the carbonate phases. The integrated application of these advanced characterization techniques provides a comprehensive understanding of the CMC process, serving as a scientific foundation for optimizing reaction conditions, enhancing carbonation efficiency, and developing more effective and sustainable CMC strategies.

## 3. Research Progress of CO_2_ Mineral Carbonation

Recently, remarkable advances have been achieved in CMC technologies, with research and application pathways spanning multiple levels, from geological mineralization to industrial utilization. The potential of CMC is not only reflected in its ability to mitigate climate change risks through long-term stable storage, but also in the generation of industrially valuable by-products, achieving multiple benefits.

Among various technological pathways, geological mineralization, as a relatively low-cost carbon reduction method, demonstrates the potential for large-scale CO_2_ storage. Concrete carbonation curing technology not only enhances the mechanical properties and durability of materials but also facilitates additional CO_2_ fixation. The mineralization of industrial solid waste, on the other hand, transforms discarded resources into value-added products by producing negative-carbon construction materials, thus achieving the dual objectives of greenhouse gas reduction and solid waste recycling.

It is noteworthy that continuously improving the added value of mineralization products is considered one of the key pathways for driving the commercialization of CMC technologies. This approach not only reduces reliance on policy incentives but also helps offset research and development investments and operational costs, thereby accelerating the large-scale deployment and industrialization of related technologies.

### 3.1. Geological Mineralization

Geological CO_2_ mineralization, also known as in situ mineral carbonation, involves injecting CO_2_ into rock formations rich in reactive minerals, such as basalt or ultramafic lithologies, where it reacts to form stable carbonates on site, thereby realizing long-term sequestration. This process is regarded as a secure carbon fixation pathway with minimal leakage risk. Once injected in supercritical, liquid, or gaseous form, CO_2_ typically undergoes slow direct carbonation reactions. In practice, CO_2_ diffuses through rock fractures, dissolves into formation water, and subsequently promotes mineral dissolution before undergoing carbonation. Rock pores and fractures provide not only migration channels for fluids but also reactive surfaces for cation release and carbonate precipitation. Consequently, the rate of in situ mineral carbonation is mainly influenced by factors including formation permeability or porosity, CO_2_ partial pressure, water chemistry, and temperature [35].

**Figure 2:**
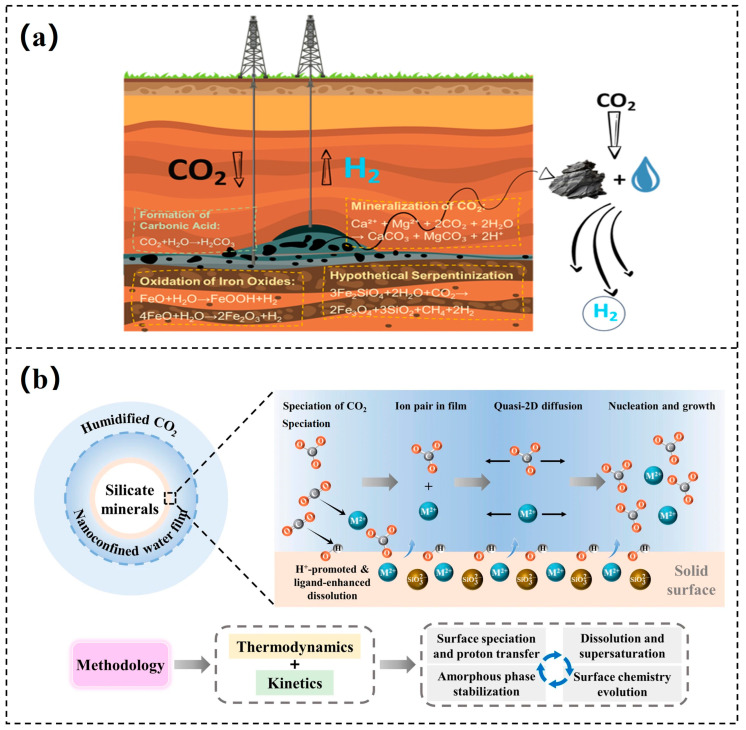
(**a**) A schematic diagram illustrating the hypothesized mechanism of CO_2_ injection and H_2_ generation [39]; (**b**) Molecular-scale mechanisms of CO_2_ mineralization in nanoscale interfacial water films [43] (licensed under CC BY 4.0; https://creativecommons.org/licenses/by/4.0/ (accessed on 22 August 2025)).

Two principal CO_2_ injection strategies have been explored: (i) direct injection of liquid or supercritical CO_2_ and (ii) injection of CO_2_ pre-dissolved in water [36]. The storage and transport of liquid or supercritical CO_2_ are commercially established; however, subsurface injection requires reservoir integrity to prevent leakage, especially in fragile or fractured strata [37]. By contrast, injecting CO_2_-rich aqueous solutions reduces leakage risks, though it demands substantial water resources to ensure adequate CO_2_ solubility [38]. In regions with freshwater scarcity, seawater or alternative fluids may serve as injection media. Ahmed et al. [39] demonstrated experimentally that injecting CO_2_ into basaltic systems can yield hydrogen concentrations reaching 0.18 wt% in the gas phase and significantly increase total inorganic carbon, confirming successful mineralization. Notably, the generated H_2_ may partially offset CO_2_ emissions, as shown in Figure 2a.

Beyond subsurface injection, numerous natural minerals-such as wollastonite, olivine, and serpentine-offer potential for surface mineral carbonation [8]. Kantzas et al. [40] reported that enhanced rock weathering in UK croplands could remove 6–30 Mt CO_2_ annually, equivalent to 45% of national emissions. Beerling et al. [41] further proposed that applying 40 t of finely ground basalt per hectare, combined with enhanced weathering, could enable US croplands to remove ~0.16–0.3 Gt CO_2_ annually by 2025. To improve the reaction activity of natural minerals, Chen et al. [42] suggested a thermal exchange process to transform magnesium silicates into highly active Ca_2_SiO_4_ and MgO, enabling rapid mineralization within hours. Similarly, Ye et al. [43] identified nanoscale interfacial water films on silicate surfaces as unique reaction media that markedly accelerate CO_2_ mineralization. In this mechanism, H^+^ in the water film promotes mineral dissolution, while rapid ion pairing and diffusion under supersaturation drive carbonate nucleation and growth (Figure 2b).

The potential of geological mineral carbonation to fix CO_2_ is immense. However, its development remains largely confined to laboratory studies and pilot-scale field tests. Continuous, low-cost, and scalable mineral carbonation technologies require further research to enable widespread deployment.

### 3.2. Mineralization of Cement and Concrete

The carbon emissions from the global production of building materials account for approximately 25% of the carbon emissions associated with fossil fuel combustion [44], making the low-carbon transformation of the construction industry crucial for achieving carbon neutrality. Traditionally, CO_2_ mineralization has been considered detrimental to the integrity of concrete structures. The reaction of carbon dioxide with hydration products can lead to reinforcement corrosion, cracking, and degradation of structural performance, accelerating the deterioration of concrete [45]. Furthermore, Tonin et al. [46] have pointed out that global warming caused by the greenhouse effect further accelerates the rate of concrete carbonation and aging, exacerbating these negative effects. However, recent studies have significantly altered this traditional understanding. Research indicates that when CO_2_ is deliberately introduced during the mixing or curing phase of concrete, it can induce a controlled mineral carbonation reaction, forming stable calcium carbonate phases within the cement matrix [47]. This process refines the pore structure, thereby enhancing the mechanical strength and durability of the concrete. As a result, CO_2_ has shifted from being viewed as a structural degradation factor to an active agent that enhances performance. Zajac et al. [48] suggested that implementing CO_2_ mineralization at various phases of cement production and the concrete lifecycle could significantly reduce carbon emissions in the cement industry [49]. Cement systems contain abundant Ca^2+^ and Mg^2+^ components, such as C_2_S and C_3_S, as well as hydration products like calcium hydroxide and C-S-H gel, all of which can react with CO_2_ to form stable calcium carbonate. This process not only captures carbon but also improves the microstructure and mechanical properties of the material. Research indicates that calcium carbonate crystals, as carbonation products, can fill pores and make the matrix denser, thus improving the strength and durability of cement-based materials [50]. Introducing CO_2_ during the concrete mixing stage can promote hydration reactions and increase early strength. However, this method faces limitations due to low CO_2_ capture efficiency and high energy consumption. To resolve this issue, Fu et al. [51] pointed out an innovative process that directly injects CO_2_ into cement slurry for mineralization, achieving up to 45% CO_2_ capture efficiency while maintaining the strength of the concrete. Currently, this engineered carbonation technology has become a hot topic in academic research and has been successfully applied on an industrial scale, achieving both carbon sequestration benefits and improvements in material performance.

**Figure 3:**
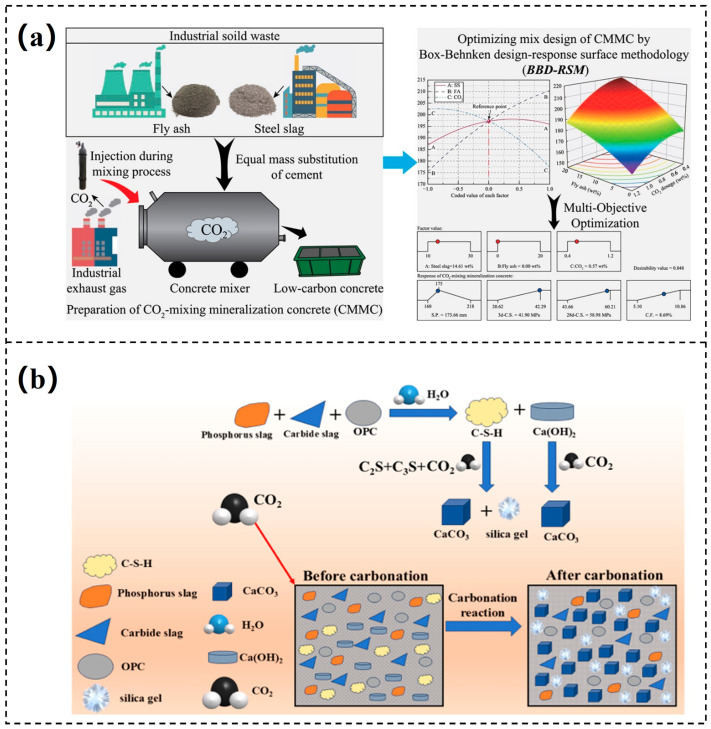
(**a**) CO_2_-mixing mineralization concrete [54]; (**b**) the diagram of carbonation mechanism of PS, CS, and OPC [60].

CO_2_ mineralization curing during the early stages of concrete or cement products (such as blocks and prefabricated panels) is also an important approach. This method accelerates hardening and CO_2_ fixation by promoting CO_2_ diffusion into the material in high-CO_2_ environments, where it reacts with hydration products to form carbonates. Research shows that this process can significantly enhance early strength within hours [52]. Compared to traditional curing methods, CO_2_ curing reduces water and thermal energy consumption and achieves higher strength in a shorter time [53]. The addition of appropriate solid waste can improve both the mineralization efficiency and mechanical properties of concrete. Xu et al. [54] found that through CO_2_ mineralization treatment, fly ash did not strengthen performance, but steel slag (14.61%) synergized with CO_2_ to yield concrete with good flowability and high strength (28-day compressive strength of 58.98 MPa), while also increasing the CO_2_ fixation rate up to 8.69%. Compared with ordinary concrete, this low-carbon concrete reduces carbon emissions by 16.9%, demonstrating significant environmental benefits, as shown in Figure 3a. Ling et al. used 100% municipal solid waste incineration residue to prepare a novel cement, which exceeded a compressive strength of 100 MPa after one day of curing, with a CO_2_ absorption rate of 11.4% [55]. Notably, the mineral carbonation treatment of construction and demolition waste can produce recycled hardened cement powder and recycled concrete aggregates to replace natural aggregates and cement clinker, thus achieving resource recycling [56]. After CO_2_ treatment, stable carbonate minerals and a three-dimensional silica gel network are formed, reducing porosity, refining pore structures, and enhancing the interface transition zone [57,58,59]. The mechanical strength and durability of recycled concrete after carbonation are comparable to those of silicate cement concrete. Liu et al. [60] studied the modification of phosphogypsum (PS) with carbide slag (CS), which effectively mitigated the delay in cement hydration caused by phosphorus and fluoride impurities. The synergistic effect of PS and CS significantly enhanced the mechanical properties and CO_2_ sequestration capacity of cement-based composite materials under carbonation curing, as shown in the carbonation mechanism diagram in Figure 3b. This provides an efficient pathway for the high-value utilization of two industrial waste and the development of low-carbon building materials, achieving both environmental and economic benefits.

### 3.3. Industrial Solid Waste Mineralization

#### 3.3.1. Steel Slag

Heavily dependent on fossil fuels, the steel sector ranks among the world’s most energy- and carbon-intensive industries, contributing significantly to industrial CO_2_ emissions and ranking as one of the largest direct sources of CO_2_ in industrial processes [61]. Steel slag, a solid waste by-product of steel production, has an annual output exceeding 100 Mt in China alone [62]. In contrast, developed countries typically achieve a steel slag utilization rate exceeding 90%, while China’s current utilization rate is only around 20% [63]. The chemical composition of steel slag is similar to that of cement clinker, containing high levels of active oxides such as CaO and MgO, which endow it with significant CO_2_ mineralization and sequestration potential. It is estimated that the global potential for carbon reduction by steel slag carbonation is approximately 138 Mt of CO_2_ annually [64].

**Figure 4:**
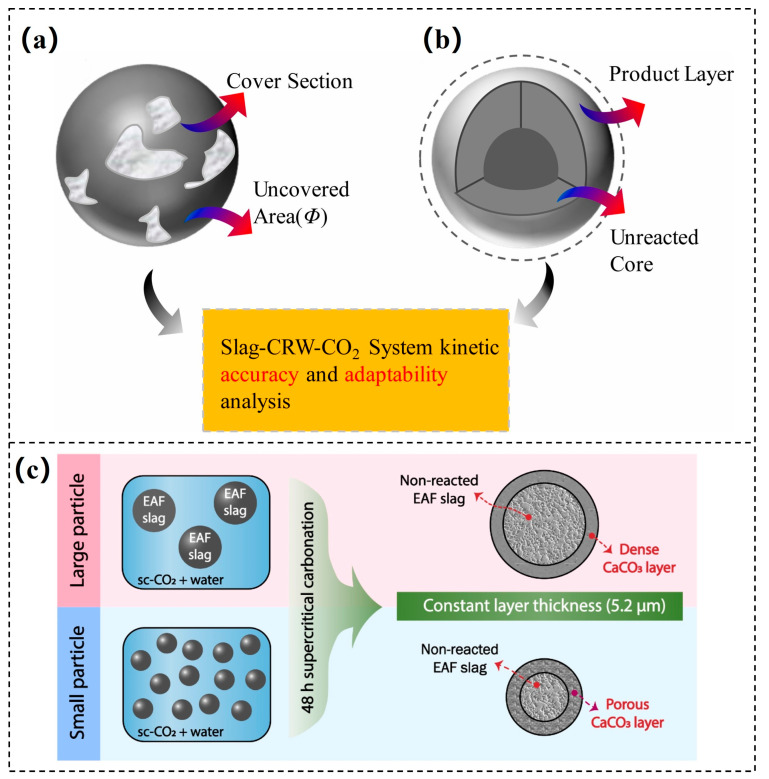
The schematic diagrams of carbonation kinetics mode: (**a**) surface coverage model [66]; (**b**) shrinking core model [66]; (**c**) the schematic diagram of carbonation with different particle sizes [70].

However, the free CaO (f-CaO) and free MgO (f-MgO) of steel slag result in volumetric expansion during hydration, limiting its direct application in the construction sector. Notably, these expansive components exhibit high reactivity when exposed to CO_2_, and their expansion risk can be effectively mitigated through mineralization treatment, thereby enhancing the value of steel slag in construction materials [65]. Apart from f-CaO and f-MgO, other calcium- and magnesium-bearing minerals in steel slag comprise approximately 40–60%, further boosting its carbonation fixation capacity. Carbonated steel slag can be utilized as a substitute for concrete aggregate, road base material, engineered wood products, or supplementary cementitious materials, offering a wide range of applications and contributing to reducing the overall carbon footprint of the construction industry [29]. The CMC process of steel slag is typically described using surface coverage models (Figure 4a) or shrinking core models (Figure 4b). Initially, steel slag particles directly interact with CO_2_ or carbonate ions, with the resulting calcium carbonate progressively forming a dense layer on the surface of steel slag. The surface coverage effect restricts the diffusion of CO_2_ and the leaching of Ca^2+^ and Mg^2+^ ions, leading to a gradual decrease or even termination of the reaction rate [53,66]. Consequently, enhancing mineralization efficiency necessitates strategies that mitigate or eliminate the surface coverage effect.

Several factors influence the CMC efficiency of steel slag, with particle size being a critical factor [67]. Studies have reported that reducing the particle size increases its specific surface area, exposing more active sites and thus accelerating the reaction rate [68,69]. Moreover, as illustrated in Figure 4c, smaller steel slag particles form a looser and porous calcium carbonate layer on the surface, which alleviates the adverse impact of surface coverage on the carbonation process [70]. In addition to particle size, process parameters such as temperature, pressure, CO_2_ concentration, and water-to-solid ratio significantly affect the carbonation efficiency of steel slag.

Additives also play an important role in the carbonation reaction [17]. Research by Deng et al. [71] demonstrated that various additives, including chelating agents, inorganic alkaline activators, and sulfates, significantly improve CO_2_ absorption efficiency. Among these, ethylenediaminetetraacetic acid (EDTA) and its salts not only accelerated the carbonation rate but also markedly enhanced the mechanical properties of steel slag blocks. Zeng et al. [72] proposed the use of sodium salicylate as a novel promoter, which was found to accelerate mineral dissolution and optimize pore structure, resulting in a 29.6% increase in compressive strength. Some advanced physicochemical-assisted techniques have played a significant role in the carbonation of steel slag. Liu et al. [73] employed microwave-enhanced acetic acid leaching to selectively extract calcium and magnesium from steel slag, and utilized the leachate to fix CO_2_, synthesizing high-value CaCO_3_ whiskers, achieving rapid CO_2_ sequestration. Despite significant progress in steel slag CO_2_ mineralization technology, existing research has predominantly focused on optimizing single factors, with a lack of systematic exploration of the coupling effects of multiple process parameters. Additionally, given the limited leaching efficiency of alkaline earth metals in steel slag, achieving deep carbonation under low-cost conditions remains a critical issue for future research [28]. Therefore, further development of steel slag CO_2_ mineralization requires interdisciplinary research and engineering exploration, from process integration and reaction kinetics control to economic and environmental sustainability assessments.

#### 3.3.2. Fly Ash

Fly ash is a major solid waste by-product generated during the combustion of fuel in coal-fired power plants, with a global annual production exceeding 10 Gt. It is currently one of the most significant industrial solid waste [17]. The chemical composition of fly ash typically contains 20–30% CaO, while the combined content of SiO_2_ and Al_2_O_3_ often exceeds 30%, endowing it with substantial carbon sequestration potential. Fly ash exhibits pozzolanic activity, allowing it to partially replace cement as a mineral admixture. However, due to the presence of alkaline components and potentially harmful substances (e.g., heavy metals), the large-scale disposal and utilization of fly ash face environmental and safety risks.

CMC technology provides a novel approach for the resource utilization of fly ash. On the one hand, the carbonation reaction can stabilize and immobilize heavy metal ions within fly ash, reducing environmental risks. On the other hand, the carbonation products can improve the strength and durability of fly ash-based materials, thereby expanding their application potential in the construction sector.

Existing studies have shown that humidity is a critical factor affecting carbonation efficiency in gas-solid carbonation processes. Liu et al. [74] synthesized a capture-mineralization composite material using modified fly ash and found that increased humidity significantly promoted reaction efficiency under direct gas-solid carbonation conditions. Agnieszka et al. [75] conducted a comparative study on the gas-solid carbonation of fly ash under dry and wet conditions, achieving a maximum carbonation efficiency of 48.14% and indicating that this process could potentially convert approximately 21% of CO_2_ emissions from coal-fired power plants into stable carbonates. Compared to gas-solid carbonation, fly ash exhibits higher carbon sequestration efficiency and faster reaction rates under direct solution carbonation conditions, making this approach more widely studied. Rushendra et al. [76] compared the two reaction pathways and found that the direct gas-solid carbonation rate was slower, while solution carbonation could achieve a maximum carbonation efficiency of 67.87%, significantly outperforming the former. Shao et al. [77] further investigated the factors influencing solution carbonation and revealed that CO_2_ pressure, water-to-solid ratio, and particle size all significantly impacted the carbonation process. Specifically, as CO_2_ pressure increased, the concentration of carbonate/carbonate ions in the solution increased, resulting in higher carbon sequestration by fly ash.

**Figure 5:**
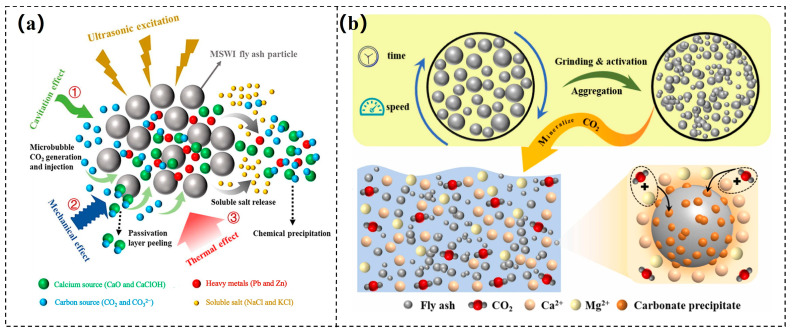
(**a**) The mechanism of fly ash ultrasonic carbonation [78]; (**b**) schematic of the CO_2_ mineralization and sequestration by treated fly ash [79].

The direct solution carbonation process can be divided into three main stages. First, CO_2_ dissolves in water, forming carbonic acid. Subsequently, carbonic acid ionizes in water, producing H^+^ that reacts with the CaO and MgO in fly ash, generating soluble Ca^2+^ and Mg^2+^. Finally, Ca^2+^ and Mg^2+^ react with CO_3_^2−^ at the surface of the fly ash, forming carbonate precipitates that accumulate on the surface. Similar to other carbonation processes involving calcium-magnesium minerals, the carbonation of fly ash can be described using surface coverage models and shrinking core models. To enhance the CO_2_ mineralization efficiency of fly ash, researchers have employed techniques such as ultrasonic treatment and grinding activation. Chen et al. [78] combined ultrasonic chemical methods with wet carbonation technology, successfully improving the carbon sequestration efficiency by 4.7%. Their study indicated that ultrasonic physical-chemical excitation could improve mass transfer efficiency and facilitate the carbonation reaction at the gas-liquid-solid three-phase interface (Figure 5a). Jiang et al. [79] utilized mechanical ball milling activation and explored the effects of milling time and milling speed on CO_2_ sequestration. Under optimal experimental conditions, the modified fly ash exhibited a CO_2_ sequestration capacity of 81.70 g CO_2_/kg, demonstrating excellent carbon sequestration ability (Figure 5b). After CO_2_ mineralization treatment, the alkalinity of fly ash decreased, and the risk of heavy metal leaching was reduced. Without compromising engineering performance, carbonated fly ash can be used as a green building material to replace 5–20% of ordinary Portland cement [80,81]. In addition, microbiologically mediated enhancement techniques show great promise in this field. Sarkar et al. [82] induced carbonation reactions through microorganisms in concrete mixed with fly ash, significantly enhancing the self-healing capacity of bio-concrete and reducing CO_2_ emissions by 39%. However, most related research has focused on laboratory-scale studies, and industrial demonstration projects remain relatively scarce. In promoting large-scale applications, it is essential to consider not only carbon sequestration efficiency, carbon content, and product structure but also factors such as economic feasibility, energy consumption, and technical difficulty. Therefore, further exploration of the continuous experimental mineralization effects of fly ash during the scaling-up process is urgently needed, along with efforts to advance its large-scale industrial application.

## 4. Applications of CO_2_ Mineral Carbonation

With the continuous advancement of fundamental research, CMC technologies are progressively moving toward larger-scale engineering demonstrations, which represent an essential step toward technological maturity and eventual commercialization. Compared with laboratory- and pilot-scale studies, engineering demonstrations not only validate process stability and economic feasibility under complex real-world conditions but also generate critical operational data and practical experience for subsequent industrial deployment. CMC technology has been successfully validated in various engineering applications, including geological CO_2_ sequestration, carbonation of cement-based materials, and carbonation of industrial solid waste. Geological CO_2_ mineral sequestration offers a safe and reliable solution for CCUS, with substantial sequestration potential, positioning it as a promising technology for large-scale CCUS deployment. This approach not only provides an effective method for addressing greenhouse gas emissions but also makes a significant contribution to global emission reduction targets. The carbonation of cement-based materials has already progressed toward commercialization, presenting a pivotal transformation strategy for the high-carbon cement industry. Given that the cement sector is a major contributor to carbon emissions, the adoption of this technology offers a viable pathway for its low-carbon transition. Moreover, the carbonation process enhances the mechanical properties and durability of cement-based materials while simultaneously reducing the industry’s carbon footprint. Industrial solid waste, which is generated in large quantities and is rich in alkaline components, holds considerable potential for CMC applications. Through this technology, such waste can be converted into high-value products, such as mineral-based materials, effectively reducing waste accumulation and facilitating the resource recovery of industrial by-products. Notably, the diverse applications of CMC technologies across different industrial sectors, such as steel, cement, power generation, and geothermal energy, highlight their broad adaptability and integration potential.

Therefore, summarizing and analyzing representative demonstration projects is of great significance for understanding the current state of technological development and identifying future optimization pathways. The following sections present several internationally recognized case studies of CMC, illustrating recent progress in process mechanisms, engineering practice, and commercialization prospects.

### 4.1. Geological CO_2_ Storage

**Figure 6:**
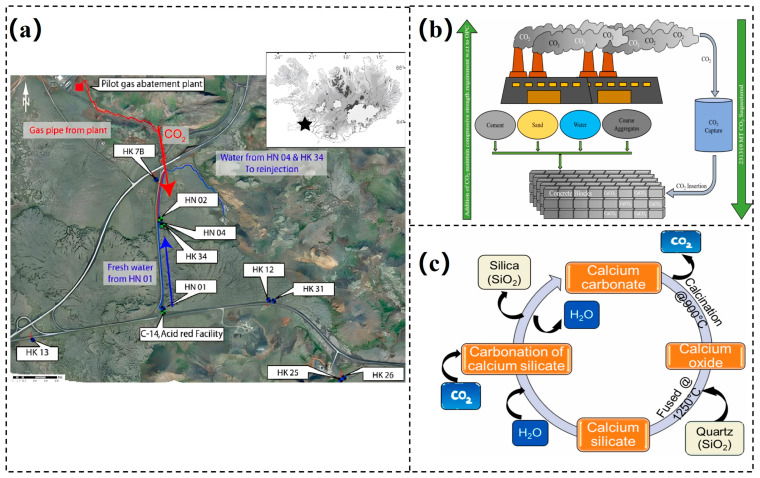
(**a**) An aerial view of CarbFix (Reykjavík, Iceland) [84]; (**b**) the chemical mechanism of CarbonCure (Dartmouth, NS, Canada) [92]; (**c**) the carbonation mechanism of Solidia [92].

The CarbFix project [83,84,85,86,87,88,89], funded by the European Union, was launched in 2012 as a pilot study at the Hellisheiði geothermal power plant in Iceland. In this project, CO_2_-H_2_S gas mixtures from the plant were dissolved in water and injected into basalt formations at a depth of ~500 m, where temperatures range between 20 and 50 °C (an aerial view of the site is shown in Figure 6a [84]). Approximately 25 t of water were required to dissolve each ton of gas. The resulting acidic solution enhanced the release of metal cations from the host rock and accelerated carbonate mineral precipitation. Isotopic and tracer monitoring indicated that more than 95% of the injected CO_2_ mineralized within two years. These findings demonstrated the efficiency and security of CO_2_ storage in basaltic formations, providing a promising pathway for large-scale carbon capture and storage (CCS).

In addition, the Wallula basalt CO_2_ sequestration demonstration project [90,91] was initiated in the United States in 2013. In this study, approximately 1000 t of liquid CO_2_ were injected into basalt formations in eastern Washington State at depths of 800–900 m. Unlike the CarbFix approach, which employed dissolution-based injection, the Wallula project injected CO_2_ directly after heating and pressurization at a rate of 40 t per day. Monitoring confirmed that within two years, the majority of the injected CO_2_ had been converted into stable carbonate minerals, with no significant leakage detected. This outcome further validated the technical feasibility of direct in situ CO_2_ mineralization in basaltic formations.

### 4.2. Carbonation of Cement-Based Materials

CarbonCure Technologies, a Canadian cleantech company, has developed a CO_2_ utilization technology [93,94,95] that injects CO_2_ directly during the concrete mixing stage. The injected CO_2_ reacts with calcium compounds in cement to form nanoscale calcium carbonate particles (the chemical mechanism is illustrated in Figure 6b [92]. This process not only achieves permanent CO_2_ sequestration but also enhances concrete compressive strength by approximately 10% while reducing cement consumption by 5–8%. Industrial trials have demonstrated that the technology exerts no adverse effects on workability, durability, or shrinkage properties of concrete, while lowering the overall carbon footprint by about 4.6%. To date, this process has been adopted by numerous concrete producers worldwide, offering both environmental benefits and economic feasibility, and providing a practical pathway toward decarbonization in the concrete industry.

Huaxin Cement Company has developed and commissioned the world’s first 100,000-ton-scale CO_2_-curing concrete production line utilizing cement kiln flue gas [96,97]. In this process, kiln exhaust containing 20–30% CO_2_ is directly applied to the curing of concrete bricks. By regulating moisture migration and carbonation reaction pathways, CO_2_ reacts with cement hydration products to form calcium carbonate crystals, thereby enabling in situ sequestration and resource utilization of industrial emissions. Operational data indicate that the production of 100 million bricks can sequester approximately 26,000 t of CO_2_, while simultaneously improving compressive strength by 13–33% and reducing water absorption by 18–36%. Moreover, the process substantially decreases natural resource consumption and the energy demand associated with steam curing. By overcoming the long-standing challenge of directly utilizing low-concentration CO_2_, this technology provides a large-scale demonstration of carbon recycling within the cement and construction materials industry.

### 4.3. Indirect Carbonation for Calcium Carbonate Production

Enhancing the value of mineralization products is one of the key drivers for advancing the commercial deployment of CMC technologies. In recent years, chemical-looping CO_2_ mineralization has achieved notable progress in the production of high-purity calcium carbonate. The SkyCycle™ process, developed by CarbonFree in the San Antonio, TX, USA, is specifically designed for the industries that are difficult to reduce the CO_2_ emission of point sources, such as steel and cement plants [98,99]. This technology employs modular reactor units that capture CO_2_ directly from flue gas and convert it into high-purity precipitated calcium carbonate (PCC) and hydrochloric acid within proprietary reactors (the system configuration is shown in [98]). Unlike conventional carbon capture methods, SkyCycle™ eliminates the energy-intensive CO_2_ purification step, enabling the direct treatment of low-concentration flue gas streams and yielding PCC with pharmaceutical-grade purity (99%).

A demonstration CCUS facility based on this technology has already been established at a steel plant in Gary, Indiana, marking the first commercial-scale application in the North American steel industry. The facility is designed to process approximately 50,000 t of CO_2_ annually, equivalent to offsetting the yearly emissions of 12,000 passenger vehicles. The resulting PCC products can be widely utilized in the paper, plastics, coatings, and high-end cosmetics industries, while the co-produced hydrochloric acid also carries considerable economic value. Collectively, these factors significantly strengthen the industrialization potential of the SkyCycle™ technology.

### 4.4. Carbonation of Industrial Solid Waste

Coupling industrial solid waste with CMC enables carbon capture and provides an effective pathway for waste valorization, offering broad application prospects. Solidia Technologies (Windcrest, TX, USA) has developed a CO_2_ mineralization-based process for producing low-carbon construction materials. The core concept is to employ industrial residues such as slag and fly ash as reactive feedstocks, into which captured CO_2_ is injected to accelerate mineralization reactions, thereby generating carbonates and silica that subsequently serve as key components of low-carbon concrete [92,100,101]. In practical applications, the process can sequester approximately 0.5 t of CO_2_ per ton of concrete produced, while simultaneously improving the compressive strength and durability of the material (the underlying carbonation mechanism is illustrated in Figure 6c [92]). At present, Solidia Technologies has partnered with several major international construction-material companies, including Cemex, to advance the engineering-scale implementation and commercialization of this technology.

## 5. Challenges and Future Prospects

In recent years, research on CMC has expanded considerably, underscoring its significant potential for reducing atmospheric CO_2_ concentrations. Currently, the research and development market for CMC is characterized by both competition and international collaboration. North America and Europe, with their strong technological innovation capabilities and supportive policy environments, have emerged as leaders in the application of this technology. Companies such as CarbonCure and Solidia have successfully commercialized cement and concrete carbonation technologies, deploying hundreds of demonstration projects globally [92,101]. These companies have not only promoted the use of low-carbon building materials but also integrated mineral carbonation technologies into the carbon reduction strategies of the construction industry.

In terms of financing, CMC has attracted substantial venture capital and industry investments. Numerous companies, through collaborations with major energy and building materials corporations, have secured funding and technical support, overcome technological bottlenecks, and entered the early stages of commercialization. CarbonCure in the U.S. and CleanCo2 in Beijing, China, for example, have advanced the large-scale application of mineral carbonation technologies through partnerships with industry, demonstrating the importance of market demand and policy support in driving technological progress [92].

Furthermore, with the increasing depth of international cooperation, the market for mineral carbonation is becoming increasingly globalized. Cross-border partnerships have not only facilitated the expansion and application of the technology but also accelerated its standardization and commercialization processes. Overall, the market for CMC is rapidly developing, with continuous technological advancements, growing policy support, and increasing capital investment. As international cooperation deepens, the market potential for these technologies is expected to be further unleashed, contributing significantly to global carbon reduction goals.

At the technical research level, CMC technology still faces fundamental bottlenecks, most notably insufficient sequestration efficiency and limited carbonation depth. During mineral carbonation, the precipitated carbonates often deposit and form a dense layer on the surface of the host minerals. This passivating layer not only suppresses the further leaching of metal ions such as Ca^2+^ and Mg^2+^ but also impedes the sustained contact of CO_2_, carbonate ions, and protons with the mineral surface, ultimately causing the carbonation reaction to stagnate [20,23,24,25]. In addition, the inherent variability in the composition and physico-chemical properties of mineral feedstocks introduces further challenges to the stability and controllability of the reaction.

To address these limitations, future research must advance along multiple dimensions. On the macroscopic level, strategies such as mechanical comminution, tuning of reaction temperature and pressure, incorporation of crystal modifiers, and optimization of curing conditions may enhance mineral surface reactivity and delay the formation of passivation layers [69,70,77]. At the microscopic scale, controlling the crystallization kinetics and polymorphs of carbonate precipitates to reduce local supersaturation and diffusion resistance represents another promising pathway. Furthermore, the development of adaptive approaches to accommodate compositional variability among different mineral feedstocks remains a pressing need. Progress in this direction may be achieved through the establishment of multiscale coupled models, the design of novel catalysts and additives, and the integration of mineralization with other CCUS technologies. Collectively, these efforts hold promise not only for improving carbonation efficiency but also for overcoming the complexity and uncertainty of raw materials, thereby advancing the large-scale deployment and long-term viability of mineral carbonation as a carbon sequestration strategy.

At the engineering application level, the mineralization process involves numerous interdependent operational variables, including reaction temperature, pressure, pH, feedstock particle size and ratio, reaction time, mixing intensity, and the type and concentration of additives. In combination, these parameters define a highly complex multidimensional space. Conventional experimental design methods, such as orthogonal arrays, exhibit limited efficiency in addressing such high-dimensional systems. They often fail to capture nonlinear coupling effects among variables, while also incurring substantial experimental costs.

To overcome these limitations, artificial intelligence (AI) has emerged as a promising tool in recent years. Leveraging historical data from laboratory experiments, pilot-scale studies, and numerical simulations, AI techniques can identify hidden patterns and establish nonlinear mapping models between process parameters and target outcomes, such as carbonation conversion, reaction kinetics, product quality, and energy consumption [102,103]. Building upon these models, AI enables multi-objective optimization, allowing for the rapid identification of optimal or near-optimal parameter sets tailored to specific goals (e.g., maximizing conversion efficiency, minimizing reaction time or energy consumption, or producing materials with desired properties). This approach not only reduces experimental workload and development costs but also significantly accelerates process innovation and enhances overall research efficiency.

Nevertheless, translating laboratory-optimized schemes into industrial applications remains challenging. The composition of industrial flue gases and solid waste feedstocks is inherently variable, while continuous mineralization processes are further influenced by fluctuations in equipment performance and external environmental conditions. These factors make it difficult to sustain reaction conditions at their optimal levels over extended periods. To address this issue, advanced control strategies such as Model Predictive Control (MPC) and Reinforcement Learning (RL) can be introduced. When combined with real-time data from online sensors, including pH, temperature, conductivity, gas concentration, and product morphology, approaches enable dynamic prediction and real-time regulation of reaction progress [104,105]. By intelligently adjusting key operational parameters such as feed rate, energy input, or pH-regulating agent dosage, it becomes possible to maintain system stability while simultaneously improving energy efficiency and resource utilization.

In addition, the widespread adoption of mineralization technologies requires a careful balance between efficiency, cost, and energy consumption. Process integration offers a promising pathway to enhance overall techno-economic performance and resource efficiency. For instance, the use of CO_2_ from ultra-low-emission coal-fired power plants for the mineralization of fly ash not only contributes to emission reduction but also promotes waste valorization. Similarly, employing surplus heat from nuclear power plants to drive mineralization reactions can significantly reduce operational costs and energy demand [106,107]. The value of process integration thus lies not only in synergistic utilization of energy and resources but also in enabling on-site treatment and valorization of industrial residues, thereby minimizing equipment retrofitting and material transport costs. Collectively, these advantages strengthen the feasibility and competitiveness of mineralization technologies for large-scale deployment.

From a commercialization perspective, enhancing the value of mineralization products is a critical pathway for advancing the industrial deployment of CO_2_ mineralization technologies. At present, the economic viability of such technologies is constrained primarily by the relatively low market value of end products, such as carbonate minerals or composite building materials, which is insufficient to offset the combined costs of CO_2_ capture, transportation, and mineralization. Consequently, process innovation and market-oriented design aimed at upgrading mineralization products from low-value bulk construction materials (e.g., aggregates and fillers) to high-performance or function-specific materials, such as high-purity industrial-grade calcium/magnesium carbonates, advanced green building materials with superior mechanical or functional properties, soil amendments, or even high-value chemical feedstocks, both broaden application scenarios and substantially improve overall techno-economic performance.

It should be emphasized, however, that the current diffusion of CO_2_ mineralization technologies remains heavily dependent on policy support. Given the constraints of technological maturity, substantial upfront investment, and cost disparities with conventional products, market forces alone are unlikely to drive widespread adoption in the near term. Therefore, a coordinated “policy–market” mechanism is essential to accelerate deployment. On one hand, financial instruments such as subsidies, tax incentives, and R&D funding can reduce the costs and risks associated with early-stage implementation. On the other hand, mandatory standards and industry regulations can generate stable demand for mineralization products. Furthermore, establishing robust carbon credit certification, measurement, and trading systems would enable sequestered CO_2_ to be converted into quantifiable and tradable assets, thereby providing a sustained revenue stream and supporting the long-term stability and scalability of mineralization technologies.

## 6. Conclusions

In summary, CMC has emerged as a durable and sustainable carbon reduction pathway, demonstrating significant potential for the permanent sequestration of CO_2_. Over the previous decades of research and practice, substantial progress has been made in elucidating its reaction mechanisms, optimizing feedstock systems, and carrying out engineering demonstrations. These advances have confirmed the feasibility and adaptability of CMC in diverse applications such as cement manufacturing, chemical production, and the valorization of industrial solid waste. Nevertheless, slow reaction kinetics, high operational costs, and challenges in large-scale deployment remain the primary bottlenecks hindering the wider adoption of this technology. Overcoming these challenges will require a deep integration of fundamental scientific research with engineering innovation, along with collaborative efforts that span multiple disciplines and industries.

Looking ahead, CMC is poised to play an increasingly critical role in the global pursuit of carbon neutrality. Beyond achieving carbon sequestration, this technology also offers the dual benefits of resource recovery and waste valorization, providing important support for the development of a circular low-carbon economy. Future research should focus on enhancing the thermodynamic and kinetic efficiency of the carbonation process, developing low-cost, high-activity reactive materials and catalytic systems, and establishing economically viable models for industrial-scale implementation. With sustained scientific innovation, strong industrial collaboration, and supportive policy measures, CMC is expected to become a vital pillar in achieving long-term climate stability and enabling a sustainable industrial transformation.

## Data Availability

No new data were created or analyzed in this study. Data sharing is not applicable to this article.
